# Complete genome sequence of the cellulose-producing strain *Komagataeibacter nataicola* RZS01

**DOI:** 10.1038/s41598-017-04589-6

**Published:** 2017-06-30

**Authors:** Heng Zhang, Xuran Xu, Xiao Chen, Fanshu Yuan, Bianjing Sun, Yunhua Xu, Jiazhi Yang, Dongping Sun

**Affiliations:** 1 Chemicobiology and Functional Materials Institute, Nanjing University of Science and Technology, Nanjing, 210094 China; 2 School of Chemical Engineering, Nanjing University of Science and Technology, Nanjing, 210094 China; 3 Department of Life Sciences, Lianyungang Normal College, Lianyungang, 222000 China

## Abstract

*Komagataeibacter nataicola* is an acetic acid bacterium (AAB) that can produce abundant bacterial cellulose and tolerate high concentrations of acetic acid. To globally understand its fermentation characteristics, we present a high-quality complete genome sequence of *K. nataicola* RZS01. The genome consists of a 3,485,191-bp chromosome and 6 plasmids, which encode 3,514 proteins and bear three cellulose synthase operons. Phylogenetic analysis at the genome level provides convincing evidence of the evolutionary position of *K. nataicola* with respect to related taxa. Genomic comparisons with other AAB revealed that RZS01 shares 36.1%~75.1% of sequence similarity with other AAB. The sequence data was also used for metabolic analysis of biotechnological substrates. Analysis of the resistance to acetic acid at the genomic level indicated a synergistic mechanism responsible for acetic acid tolerance. The genomic data provide a viable platform that can be used to understand and manipulate the phenotype of *K. nataicola* RZS01 to further improve bacterial cellulose production.

## Introduction

Acetic acid bacteria (AAB) are a group of microorganisms that belong to the family *Acetobacteraceae* of the class *Alphaproteobacteria*. These bacteria are widely found on fruits, flowers, and rotten food^1^. Their metabolic uniqueness has been utilized in the industrial production of sorbose, vitamin C, dihydroxyacetone, d-gluconic acid, and bacterial cellulose (BC)^2–5^. Furthermore, AAB can also act as fermentative organisms, therefore, they are applied in beer, wine, and vinegar production^6, 7^. A remarkable feature of these strains is their ability to survive under extreme environments, such as high sugar concentrations and low pH values, which makes AAB suitable for various industrial applications^8^.

Currently, AAB include 14 genera, namely *Acetobacter*, *Gluconobacter*, *Gluconacetobacter*, *Komagataeibacter*, *Granulibacter*, *Asaia*, *Acidomonas*, *Kozakia*, *Swaminathania*, *Saccharibacter*, *Neoasaia*, *Tanticharoenia*, *Ameyamaea*, and *Neokomagataea* ^1^. Classification within the *Acetobacteraceae* depends on the ubiquinone type, including the Q-9 and Q-10 types. *Acetobacter* species use the Q-9–type ubiquinone, whereas the AAB genera of *Gluconacetobacter* and *Gluconobacter* contain mainly the Q-10–type ubiquinone^9^. The genus *Komagataeibacter*, a gram-negative, obligately aerobic and rod-shaped acidophilic organism, was initially proposed by Yamada in 2012, with 12 other strains on the basis of their taxonomic characteristics^10^. *Komagataeibacter* gen. nov. originated from the *Gluconacetobacter xylinus* (previously named *Acetobacter xylinus*) group, which is first separated from the *Gluconacetobacter liquefaciens* group at the genus level. These 2 groups are significantly different in phylogenetic and phenotypic characteristics^11^. Thus far, 66 complete genomes of *Acetobacteraceae* have been published in NCBI databases (http://www.ncbi.nlm.nih.gov/), most of which just present draft genome sequences. The genomes of 3 species of *Komagataeibacter*, *K. xylinus* E25, *K. hansenii* ATCC 23769, and *K. medellinensis* NBRC3288, have been sequenced completely. However, draft genomes are available for *K. europaeus* LMG18494, *K. intermedius* AF2, *K. rhaeticus* AF1, *K. kakiaceti* JCM 25156, and *K. oboediens* 174Bp2^12, 13^.

Generally, *Komagataeibacter* species possess the ability to secrete exopolysaccharides (EPS), especially BC^14^. Notably, *K*. medellinensis NBRC3288 is the only cellulose-nonproducing member of *Komagataeibacter* species, isolated from vinegar^15^. BC has been the focus of research owing to its excellent properties, such as high purity, predominant three-dimensional structure, high degree of crystallinity, and superior biocompatibility^16–18^. In view of these qualities, it is utilized commercially in the development and production of health food, flexible electrodes, acoustic speakers, and tissue scaffolds^16, 19–21^.

*K. nataicola* RZS01 originates from rotten apples and is regarded as the model organism for BC production, owing to its high resistance to ethanol and acetic acid, which makes it an ideal strain for industrial applications^22, 23^. In this study, we present the complete genome sequence and sequence analysis of *K. nataicola* RZS01 to obtain genetic information for further insights into its biochemical features. Comparison of *K. nataicola* RZS01 with other AAB strains will address the unique functional characteristics of this strain, as well as the common properties within the *Acetobacteraceae*. Furthermore, the genome data will also provide a rich resource for guiding future research in industrial applications.

## Results

### General features of the *K. nataicola* RZS01 genome

**Figure 1:**
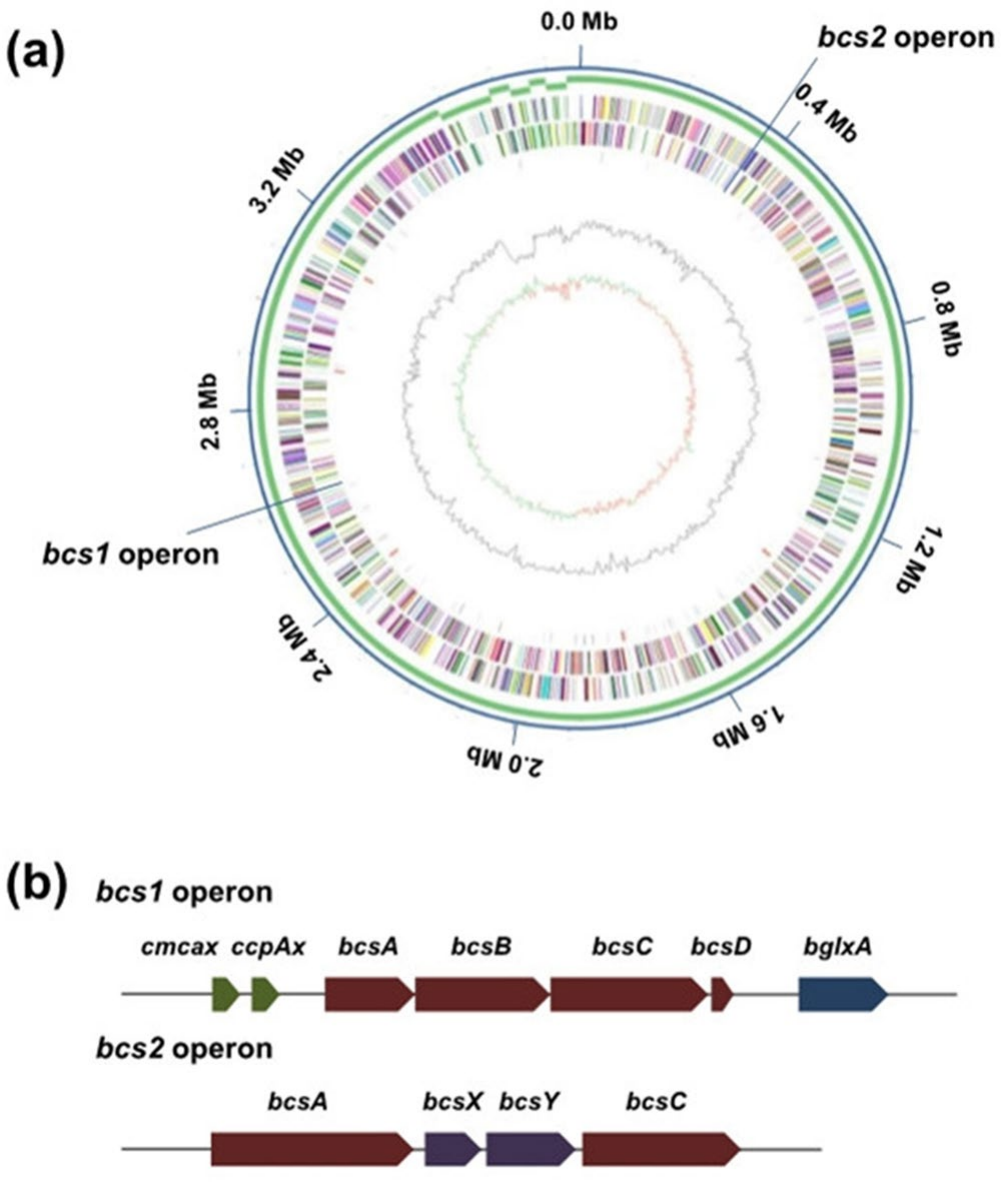
Overview of *K. nataicola* RZS01 genome. (**a**) The circles represent (from the outside to the inside): circle 1, DNA base position (bp); circle 2, contig components; circle 3, protein-coding regions transcribed clockwise; circle 4, protein-coding regions transcribed anti-clockwise; circle 5, distribution of genes encoding ncRNA (black), tRNA (blue) and rRNA (red); circle 6, G + C content; circle 7, GC skew. (**b**) The compositions of 2 *bcs* operons, which differ from each other in gene content.

The complete genome of RZS01 is composed of 1 circular chromosome of 3,485,191 bp with a G + C content of 61.49%, and 6 plasmids ranging in size from 25,766 bp to 102,282 bp (Table 1; Fig. 1). Gene prediction and annotation of the RZS01 genome resulted in 3,609 open reading frames. The number of genes in the plasmids occupies 6.5% of the total genes in the genome, and genes with predicted function are assigned to 68.86% of the genome (2,485 genes). Furthermore, 5 ribosomal RNA operons (16S-23S-5S) were detected and 62 tRNA genes were predicted. From the genome data, we also identified 66 transposase genes, which might be responsible for the genetic instability leading to deficiencies in various physiological properties as reported in other AAB^24^.

**Table 1:** General features of the *Komagataeibacter nataicola* RZS01 genome.

Features	
Size of the chromosome (bp)	3,485,191
Size of the plasimds (bp)	102,282 (pKNA01)
	39,914 (pKNA02)
	38,682 (pKNA03)
	37,912 (pKNA04)
	30,554 (pKNA05)
	25,766 (pKNA06)
DNA scaffolds	7
G + C content (%)	61.49
Total genes	3,609
Protein coding genes	3,514
RNA genes	95
Genes with function prediction	2,485

**Figure 2:**
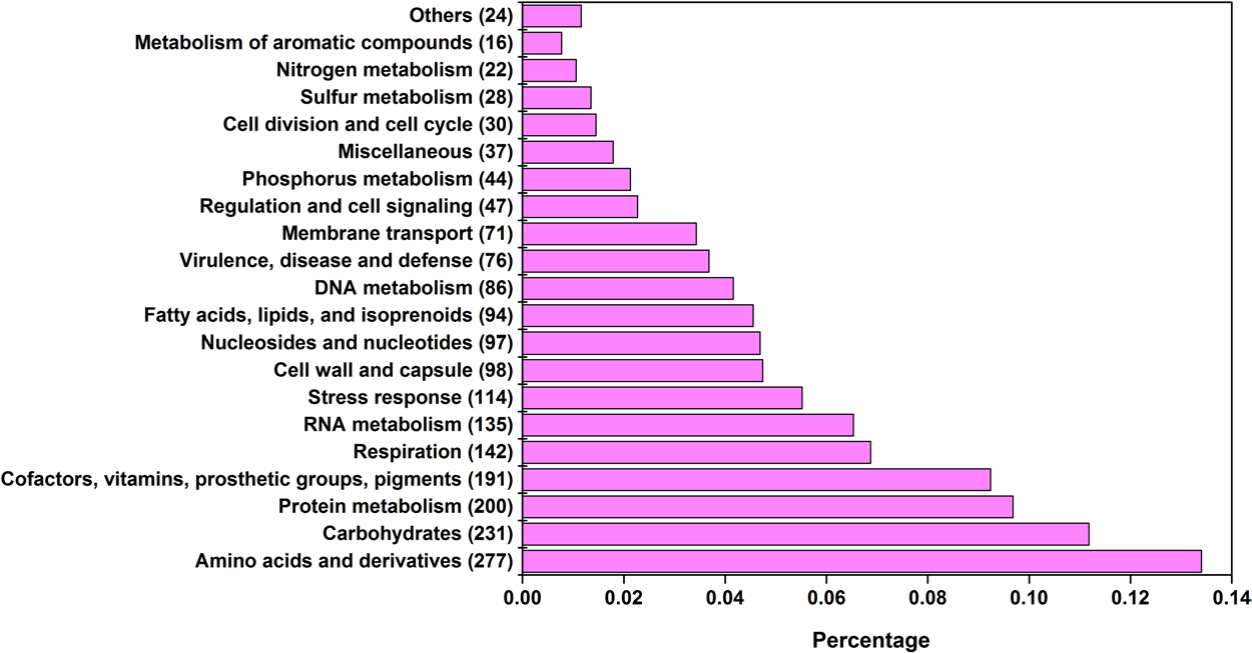
Subsystem category distributions of RZS01 proteins.

The complete genome has a total of 3,514 putative coding sequences, among which 2,485 are assigned a putative function, and 1,029 encode hypothetical proteins. The 6 plasmids have 228 coding DNA sequences (CDSs), of which approximately 47.8% encode hypothetical proteins. The remaining 42 CDSs encode mobile element proteins. Figure 2 summarizes the distribution of RZS01 proteins among 21 functional groups. Statistically, nearly 38% CDSs are completely uncharacterized, which is similar to the proportion of unassigned CDSs in some other sequenced bacterial genomes, such as in *Escherichia coli* (40%), *Haemophilus influenzae* (43%), and *Mycoplasma genitalium* (32%)^25^. The 2 largest functional groups contain 277 and 231 proteins involved in carbohydrate and amino acid metabolism, respectively, which correspond to the physiological functions in RZS01.

### Phylogenetic analysis and comparative genomics

**Figure 3:**
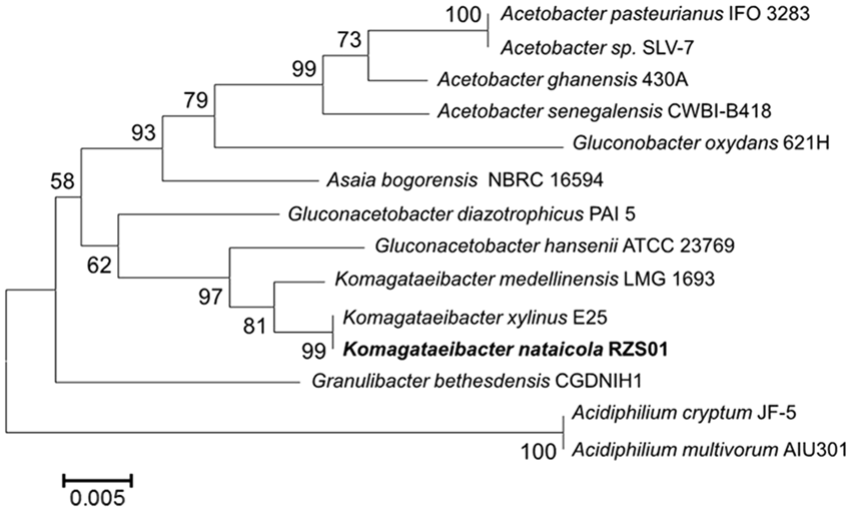
A phylogenetic tree based on 16S rRNA sequences constructed by the neighbour-joining method of MEGA5.1.

Analysis of the most conservative sequence in the process of evolution, the 16S rRNA gene, is widely used in taxonomic resolution in bacteria. To ascertain the phylogenetic positions of RZS01 in *Acetobacteraceae*, a phylogenetic tree was constructed using 16S rRNA gene sequences (Fig. 3). The phylogenetic tree summarizes 13 other *Acetobacteraceae*, the genomes of which have been completely sequenced. The tree suggests that RZS01 is most closely related to *Komagataeibacter xylinus* E25, *Komagataeibacter medellinensis* LMG 1693, and *Gluconacetobacter hansenii* ATCC 23769. Furthermore, comparative analysis of RZS01 in relation to other members of the *Acetobacteraceae* family reveals that this strain has 36.1% shared genes with the human pathogen *Granulibacter bethesdensis* CGDNIH1 (Table 2). Approximately 40% of the genes are in common with members of the genus *Acidiphilium*. Thus, RZS01 has more genes in common with *Komagataeibacter* species than with *Gluconacetobacter*, which corresponds with the phylogenetic relationship.

**Table 2:** Comparative analysis of *Komagataeibacter nataicola* RZS01. with members of the *Acetobacteracea* family.

Acetic acid bacterium strain	Number of shared genes	Percentage of shared genes
*Gluconacetobacter diazotrophicus* Pal5	5031	72.0
*Komagataeibacter medellinensis* NBRC 3288	4742	75.0
*Acetobacter senegalensis* 108B	4339	61.7
*Komagataeibacter xylinus* E25	4335	65.0
*Komagataeibacter hansenii* ATCC 23769	3592	52.4
*Acetobacter pasteurianus* IFO 3283	3216	51.1
*Acidiphilium multivorum* AIU301	3098	44.5
*Gluconobacter oxydans* 621H	2993	50.3
*Acetobacter sp*. SLV-7	2893	48.1
*Acetobacter ghanensis* 430A	2774	46.0
*Asaia bogorensis* NBRC 16594	2742	43.7
*Acidiphilium cryptum* JF-5	2656	39.4
*Granulibacter bethesdensis* CGDNIH1	2146	36.1

### Cellulose production

*Acetobacteraceae* are characterized by their ability to synthesize high yields of cellulose. Maria José Valera *et al*.^26^ investigated the cellulose-producing ability of 77 strains of 35 species of AAB, of which 19 demonstrated high yields of cellulose after cultivation. Species of the genus *Komagataeibacter* are considered to be the main producers of BC, particularly strains of the species *K. xylinus*, *K. hansenii*, *K. swingsii*, *K. rhaeticus*, and *K. nataicola* ^27, 28^. In fact, *K. xylinus* is considered a model microorganism for BC production^29, 30^.

On the RZS01 chromosome, we found the full set of cellulose synthase genes in the *bcs1* operon, which comprised the cellulose synthase genes *bcsA1* (B0W47_12635), *bscB1* (B0W47_12640), *bcsC1* (B0W47_12645), and *bcsD1* (B0W47_12650), as well as *cmcax* (B0W47_12625), *ccpAx* (B0W47_12630) upstream, and *bglxA* (B0W47_12655) downstream. *cmcax* encodes endo-β-1,4-glucanase, and *bglxA* encodes β-glucosidase, both of which have been suggested to assist cellulose biosynthes is by hydrolysing tangled glucan chains when there is a failure in chain arrangement, and all these genes are necessary for cellulose production^31^. In addition to the *bcs1* operon, we found an additional *bcs operon* (*bcs2*) at the genomic position 363284–373685, and it differs in structure from the *bcs1* operon. The *bcs2* operon is composed of *bcsA* and *bcsC*, with 2 additional genes homologous to *bcsX* and *bcsY* in the middle of *bcsA* and *bcsC*. The protein encoded by *bcsA* contains the catalytically active subunit with a PilZ domain, which is responsive to c-di-GMP^32^. An additional *bcsA* (*bcsA3*) is also located on the reverse strand. Unlike the *bcsA1* gene, the *bcsA2* and *bcsA3* genes do not contain a PilZ domain.

Generally, AAB possess the ability to secrete other polysaccharides, including capsular polysaccharides, lipopolysaccharides, and water-soluble EPS. One of the most studied polysaccharides is a xanthan gum-like EPS, named ‘acetan’, the structure of which is shown in Supplementary Information. The chemical repeat unit consists of a cellobiose unit solubilized by attachment of a charged pentasaccharide sidechain to one of the glucose residues^33, 34^. Genetic analysis of RZS01 shows that the biosynthesis of acetan occurs via a pathway similar to that for BC biosynthesis. Because it is the composite of different monosaccharides, 2 CDSs are responsible for encoding UDP-glycosyl transferase (B0W47_01855, B0W47_03955, and B0W47_12635), GDP-mannosyl transferase (B0W47_09075, and B0W47_11925), and phosphomannose isomerase (B0W47_06245). The enzymes UDP-glycosyl transferase and GDP-mannosyl transferase transfer a glucose and mannose residues from UDP-Glc and GDP-Man, respectively, to one of the intermediates of acetan^35^. However, not all genes from the gum operon are present in RZS01. Only 2 CDSs (B0W47_14415 and B0W47_14460) represent the genes of *gumK* and *gumH*, respectively. The genes for *gumB*, *C*, *D*, *E*, *G*, *I*, *J*, and *M* are absent in the RZS01 genome.

### Regulation of cellulose biosynthesis

BC biosynthesis is regulated by the expression of *bcs* genes, which appear to be expressed in response to the second messenger cyclic-di-GMP (c-di-GMP)^36^. An earlier study revealed that cellulose biosynthesis could be stimulated almost 100-fold by c-di-GMP^37^. c-di-GMP is produced from 2 molecules of GTP by diguanylate cyclases (DGCs) and is broken down into 5′-phosphoguanylyl-(3′-5′)-guanosine by specific phosphodiesterases (PDEs). DGC activity is associated with the GGDEF domain, whereas PDE activity is associated with EAL or HD-GYP domains; all these domains are essential for enzymatic activity^38^. We found 3 *cdg* operons (*cdg1*, *cdg2*, and *cdg3*) containing a c-di-GMP PDE gene followed by a DGC gene. Besides, 4 standalone c-di-GMP PDEs (*pde1–4*) and 1 standalone DGC are present in the genome, which share 32*–*40% amino acid sequence identity with each other. These multiple genes together control the intracellular c-di-GMP levels.

### Genes for substrate metabolism

On the basis of the analysis of gene annotation for the complete genome, *K. nataicola* RZS01 possesses various transporters for the uptake of substrates and ions. We identified 49 ABC transporters, 10 symporters, and 47 permeases, which are responsible for the transportation of sugars (e.g. glucose and ribose), polyols (e.g. mannitol and sorbitol), sugar acids (e.g. gluconate and acetate), amino acids, purines, pyrimidines, phosphate, sulphate, NH_4_^+^, and metal ions. In addition, some components of the phosphotransferase system are also found. As the most widely used substrate for industrial production, glucose could be taken up by a sugar symporter (B0W47_14370). Owing to the lack of the gene encoding phosphofructokinase (EC 2.7.1.11), RZS01 has an incomplete Embden-Meyerhof-Parnas pathway, as has been reported in *Acetobacter pasteurianus* 386B^6^, *Gluconacetobacter diazotrophicus* Pal5^39^, and *Gluconobacter oxydans* 621H^2^. However, the genes encoding the enzymes of the pentose-phosphate (PPP) pathway are all found, suggesting that glucose might be degraded via the PPP pathway. Carbohydrates are generally metabolized by dehydrogenases and isomerases after phosphorylation by specific kinases. In addition, RZS01 possesses an orthologous cluster for gluconate utilization similar to the *GntU*, *GntK*, and *GntR* cluster found in *E.coli* ^40^. However, RZS01 does not contain a *GntK* gene, which encodes gluconate kinase. As for disaccharides, such as sucrose and lactose, substrates can be utilized after being decomposed to monosaccharides. RZS01 contains levansucrase (B0W47_08430), which converts sucrose into glucose and fructose for further oxidation, suggesting that this strain can take up sucrose from the environment to use it as a carbon source. Sucrose is able to adjust the osmotic pressure of the medium, which has been verified by Velasco-Bedrán^41^.

Genome analysis illustrated that RZS01 possesses all the genes catalysing metabolic pathways involved in the *de novo* synthesis of all nucleotides, amino acids, phospholipids and many vitamins. Ammonia, which participates in the activity of glutamate synthase (B0W47_13175 and B0W47_13180) and glutamine synthase (B0W47_07980), can be taken up by a specific ammonia transporter (B0W47_07675).

### Membrane-associated primary dehydrogenases and the respiratory chain

*K. nataicola* RZS01 contains several genes that encode membrane-associated primary quinone reductases coupling substrate oxidation with quinone reduction, which can be classified into 2 groups (Table 3)^2^. The first group of dehydrogenases depends on the cofactor pyrroloquinoline quinine (PQQ). The most representative dehydrogenases are PQQ-dependent alcohol dehydrogenase (PQQ-ADH) and aldehyde dehydrogenase (PQQ-ALDH), which convert alcohol into acetaldehyde and acetaldehyde into acetate, respectively. The genes encoding PQQ-ADHs are present as 8 copies distributed throughout the chromosomes, and just is localized in the membrane. Moreover, RZS01 possesses 1 membrane-bound PQQ-ALDH, whereas 5 other copies are dispersed in the cytoplasm. Furthermore, the genome of RZS01 contains glucose dehydrogenase and glycerol dehydrogenase.

**Table 3:** Primary membrane-bound dehydrogenases in *K. nataicola* RZS01.

Family	Cofactor	Genes
Glucose dehydrogenase	PQQ	B0W47_01230, B0W47_02520, B0W47_10950
Glycerol dehydrogenase	PQQ	B0W47_01005, B0W47_01010
Alcohol dehydrogenase	PQQ	B0W47_13410
Aldehyde dehydrogenase	PQQ	B0W47_16410
2-Keto-d-gluconate dehydrogenase	FAD	B0W47_11030, B0W47_11035, B0W47_11040
Gluconate 2-dehydrogenase	FAD	B0W47_05395, B0W47_05400, B0W47_05405, B0W47_13405

### Adaptation to extreme conditions

*K. nataicola* RZS01 possesses the ability to tolerate low pH and high concentrations of organic acid in the environment^42^. One strategy for this tolerance is that RZS01 has a cytosolic acetate-assimilating detoxification pathway, whereby acetate is first converted into acetyl-CoA, which is catalysed by acetyl-CoA synthetase (*acn*; B0W47_06505). The product is further oxidized by the citric acid cycle to water and carbon dioxide. Overoxidation occurs in *Acetobacter* and *Gluconacetobacter* but not in *Gluconobacter*, which exhibits relatively weak acetic acid resistance^8, 43^. The second mechanism for acid tolerance may involve an ABC transporter gene, *aatA* (B0W47_06270), which functions as an efflux pump of acetic acid^44^. In another similar efflux mechanism, a proton motive force-dependent efflux system occurs in the cytoplasmic membrane, which is capable of exporting acetic acid and is dependent on the proton motive force but not on ATP^45^. Besides, there is also an operon involved in high resistance to acetic acid, a trait that can be a suitable target for enhancement by breeding. This operon is composed of the genes *groE* (B0W47_13360 and B0W47_13365), *groS* (B0W47_00055), and *groL* (B0W47_02405, B0W47_03785, and B0W47_09650), and the overexpression of the *groESL* operon increases acetic acid resistance in *Acetobacter* ^46^. A previous thorough genome analysis revealed the presence of a gene cluster encoding a urease (*ureDABCEFG*; B0W47_16420, B0W47_16425, B0W47_16430, B0W47_16435, B0W47_16440, B0W47_16445, and B0W47_16450), a urea transporter (*urtABCDE*; B0W47_16475, B0W47_16480, B0W47_16485, B0W47_16490, and B0W47_16495), and an allophanate hydrolase (B0W47_14240, B0W47_14245, B0W47_16295, and B0W47_16300). These proteins transport urea and catalyse it to ammonia, which enables the survival of RZS01 in an acidic environment, such as in vinegar fermentation. This mechanism is also present in *A. pasteurianus* 386B and *G. bethesdensis* CGDNIH1 but is not widespread among AAB strains^6, 47^.

## Discussion

*K. nataicola* is widely distributed in nature and displays strong abilities of producing BC and tolerating acetic acid. In this study, we sequenced, annotated, and analysed the complete genome sequence of *K. nataicola* RZS01 and found that RZS01 possesses a 3.5-Mb chromosome and 6 plasmids. The global overview of all genes provided comprehensive insights into the metabolic features, including the uptake of different substrates and intolerance of acidic stress during BC production.

Comparative genome analysis of 13 AAB revealed that the genome of *K. nataicola* RZS01 is most closely related to that of *K. medellinensis* NBRC 3288, with 75.0% shared genes, which is congruent with the phylogenetic relationship. On the basis of the genomic sequence of RZS01, we can now more extensively describe the process of cellulose biosynthesis and the physiological basis of the underlying pathways in this organism. For other AAB species whose genomes have been sequenced, 2 *bcs* operons are present in RZS01, which may explain the high cellulose productivity observed. However, the regulation of AcsAB activity by c-di-GMP may also play important roles. We identified 3 *cdg* operons containing a DGC and a c-di-GMP PDE. Four standalone c-di-GMP PDEs and 1 standalone DGC are also present in the genome. This regulatory mechanism has also been found in other bacteria^38^.

*K. nataicola* RZS01 contains many membrane-bound dehydrogenases, which are responsible for the assimilation of substrates and contribution to acetic acid resistance, resisting the presence of high concentrations of acetic acid. The metabolism of several amino acids, such as threonine, glycine, and ornithine, produces a large amount of ammonia, which decreases the intracellular pH value. Certain other mechanisms also participate in acetic acid tolerance, including alcohol overoxidation, an acetate assimilation-related mechanism, an ABC transporter, and a proton motive force-dependent efflux system.

In summary, we uncovered global insights into BC production and acidic resistance mechanisms by genome analysis. These results provide useful information for further studies on evolution and genetic variation in *Komagataeibacter* and provide a valuable resource for biological research. Furthermore, comparative genomics analysis and functional genomics analysis can also be performed to trace the origin and evolution of this organism.

## Methods

### Bacterial strain and isolation of chromosomal DNA

*K. nataicola* RZS01 (CGMCC number 10961) used in this study was isolated from rotten apples^48^. For genomic DNA extraction, RZS01 was grown from a single colony in HZ medium, containing 20 g·L^−1^ glucose, 6 g·L^−1^ ammonium sulphate, 1 g·L^−1^ monopotassium phosphate, 0.4 g·L^−1^ magnesium sulphate, 3 g·L^−1^ peptone, 2.25 g·L^−1^ yeast extract, and 0.4 g·L^−1^ carboxymethylcellulose sodium at 30 °C for 24 h. The produced cellulose was digested by adding 0.2% cellulase and maintained at 30 °C for 2 h. After the cells were harvested, chromosomal DNA was isolated using the QiaAmp DNA Mini kit (Qiagen, Valencia, Germany) following the manufacturer’s instructions. The quality of the DNA was evaluated using a Nanodrop Spectrophotometer (Thermo Fisher Scientific, Waltham, MA, USA) after isolation.

### Genome sequencing and assembly

The wholegenome of *K. nataicola* RZS01 was sequenced at HangZhou GeneRui Biotechnology Co., Ltd. (Hangzhou, China) using a PacBio RS DNA sequencer. The average insert size was 550 bp. Data from 515-Mb paired-end reads were delivered by Baseclear as 2 fastq files. The FastQC procedure was used to verify the raw read data, and QC metrics including insert-sizes, mapped reads, unmapped reads and reads that align with a deviated pattern were examined^49^. The sequencing errors were discarded using the error-correction module of Allpaths-LG. For single-molecule real-time sequencing (SMRT), a SMRT bell DNA template library with an insert size of 2~40 kb was prepared. Subsequently, the fragmented DNA was end-repaired and ligated into hairpin adapters. Sequence reads have been deposited in the ENA Sequence Read Archive (EMBL: ERS550016). Sequencing reads were corrected using the HGAP pipeline^50^. Genomic sequences were assembled *de novo* using PacBio analysis RS_HGAP_Assembly.3 (Pacific Biosciences, Menlo Park, CA, USA).

### Bioinformatics analysis

Automatic gene prediction and annotation were performed using Glimmer3.0. The genome was visualized using Circos (http://circos.ca/tutorials/lessons/). 16S rRNA phylogeny was constructed by the neighbour-joining method of MEGA5.1 at default settings. Metabolic enzymes were identified from UniProt (http://www.uniprot.org/) and BLASTp analysis (https://blast.ncbi.nlm.nih.gov/Blast.cgi) was used to identify orthologues in the genomes.

### Nucleotide sequence accession numbers

The accession numbers of the complete sequences of *K. nataicola* RZS01 determined in this study and of the 6 plasmids from NCBI can be found in GenBank (http://www.ncbi.nlm.nih.gov) under the accession no. CP019875 to CP019881.

## Electronic supplementary material

Supplementary information
