# Supplementary material for: Complete genome sequence of the cellulose-producing strain Komagataeibacter nataicola RZS01
Source: Sci Rep. 2017 Jun 30;7:4431. doi: 10.1038/s41598-017-04589-6 (PMC5493696; doi:10.1038/s41598-017-04589-6)
Supplement: Supplementary file 1 — Supplementary information [file 41598_2017_4589_MOESM1_ESM.doc]

Supplementary Information

**Complete genome sequence of the cellulose-producing strain** ***Komagataeibacter nataicola* RZS01**

**Heng Zhang**^1,2^**, Xuran Xu**^1,2^**, Xiao Chen**^1,2^**, Fanshu Yuan**^1,2^**, Bianjing Sun**^1,2^**, Yunhua Xu**^3^**, Jiazhi Yang**^1,2✻^ **& Dongping Sun**^1,2^**^†^**

^1^ Chemicobiology and Functional Materials Institute, Nanjing University of Science and Technology, Nanjing, 210094, China

^2^ School of Chemical Engineering, Nanjing University of Science and Technology, Nanjing, 210094, China

^3^ Department of Life Sciences, Lianyungang Normal College, Lianyungang, 222000, China

^✻^Corresponding author, Jiazhi Yang, e-mail: jiazhiyang@sina.com

^†^ Corresponding author: Dongping Sun, e-mail: sundpe301@163.com


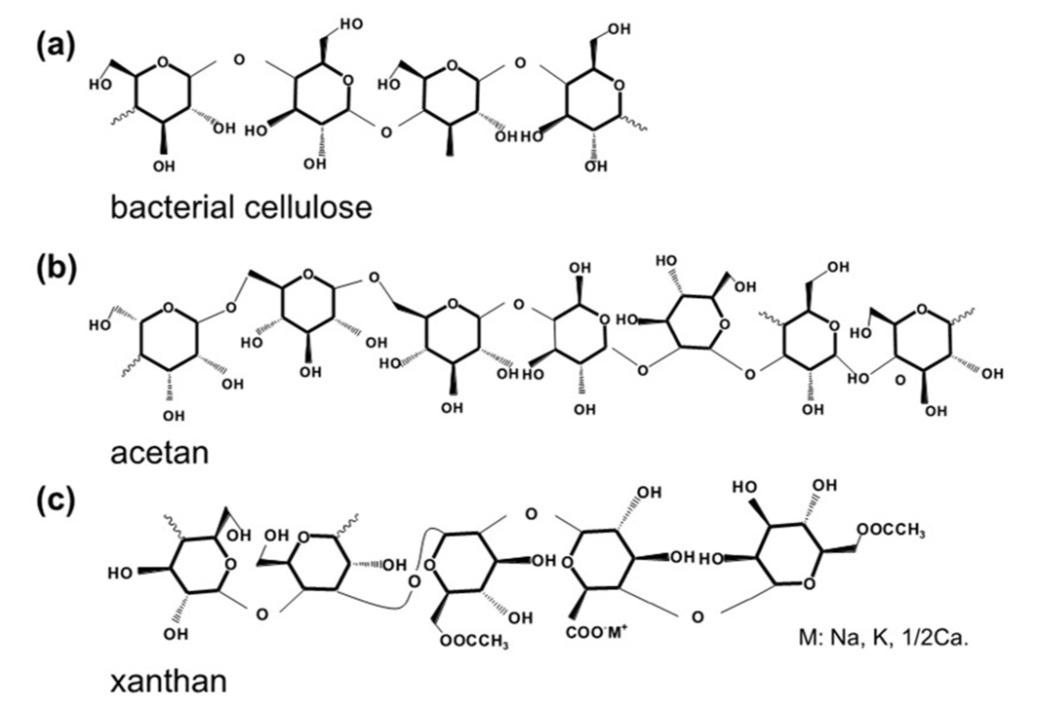


**SI 1: Structure of the extracellular polysaccharide of *K. nataicola*.**
